# Stepwise Translational Validation of the Screening Hit Desipramine Reveals Limits of Fibroblast-State Modulation in Lung Fibrosis

**DOI:** 10.3390/cells15151344

**Published:** 2026-07-27

**Authors:** Georgios-Dimitrios Panagiotidis, Stefano Rivetti, Manuela Marega, Afshin Noori, Elie El Agha, Malgorzata Wygrecka, Peter Braubach, Raffaella Klima, Luca Braga, Saverio Bellusci

**Affiliations:** 1 Department of Medicine V, Internal Medicine, Infectious Diseases and Infection Control, Universities of Giessen and Marburg Lung Center (UGMLC), German Center for Lung Research (DZL), Justus-Liebig University Giessen (JLU), 35392 Giessen, Germanyelie.el-agha@innere.med.uni-giessen.de (E.E.A.); 2 Cardio-Pulmonary Institute (CPI), 35392 Giessen, Germany; 3 Institute for Lung Health (ILH), 35392 Giessen, Germany; 4 Department of Medicine II, Internal Medicine, Pulmonary and Critical Care, Universities of Giessen and Marburg Lung Center (UGMLC), German Center for Lung Research (DZL), Justus-Liebig University Giessen (JLU), 35392 Giessen, Germany; 5 Department of Biochemistry, Faculty of Medicine, Universities of Giessen and Marburg Lung Center, Friedrichstrasse 24, 35392 Giessen, Germany; malgorzata.wygrecka@innere.med.uni-giessen.de; 6 Institute for Pathology, Hannover Medical School, 30625 Hannover, Germany; 7 International Centre for Genetic Engineering and Biotechnology (ICGEB), 34139 Trieste, Italy; 8 Department of Respiratory and Critical Care, Zhejiang-Germany Joint Laboratory on Lung Injury Repair and Regeneration, Quzhou People’s Hospital, The Quzhou Affiliated Hospital Wenzhou Medical University, Quzhou 324000, China; 9 Laboratory of Extracellular Matrix and Regeneration, Justus-Liebig University Giessen (JLU), Cardio-Pulmonary Institute (CPI), Member of the German Center for Lung Research (DZL), Institute for Lung Health (ILH), 35392 Giessen, Germany

**Keywords:** idiopathic pulmonary fibrosis, desipramine, fibroblast plasticity, myofibroblast, lipofibroblast, alveolosphere, precision-cut lung slices, drug repurposing

## Abstract

**Highlights:**

**What are the main findings?**

- Desipramine altered the profibrotic phenotype of TGF-β-stimulated WI-38 fibroblasts, with reduced myofibroblast-associated markers and increased lipid-associated staining.
- The compound showed partial activity in a fibroblast-supported alveolosphere model but failed to show consistent antifibrotic effects in human fibrotic PCLS and bleomycin-injured mice.

**What are the implications of the main findings?**

- Desipramine should be regarded as a context-dependent fibroblast-state modulator rather than a robust antifibrotic lead.
- These findings emphasize that fibroblast-based screening alone may overestimate antifibrotic potential and should be complemented by validation in more complex models.

**Abstract:**

Idiopathic pulmonary fibrosis (IPF) is a progressive and fatal interstitial lung disease with limited treatment options. Depression and anxiety are common comorbidities in patients with IPF, and emerging evidence suggests that neuroactive pathways may also influence fibrotic remodeling. On this basis, we investigated desipramine, a tricyclic antidepressant, as a potential modulator of fibroblast state in lung fibrosis. Desipramine was identified in an FDA-approved compound screen as a pro-lipogenic hit in TGF-β-stimulated fibroblasts and was subsequently evaluated across a stepwise validation pipeline of increasing biological complexity. In WI-38 fibroblasts, desipramine was well tolerated at 10 μM and reduced myofibroblast-associated features while increasing lipid-associated staining. In a fibroblast-supported alveolosphere assay, desipramine altered qualitative organoid clustering and changed the transcript levels of specific mesenchymal markers under profibrotic stimulation, whereas direct treatment of MLE-12 epithelial cells did not elicit a consistent response. While desipramine demonstrated pro-lipogenic and anti-myofibroblastic phenotypic shifts in reductionist 2D cultures, these effects failed to translate robustly into complex 3D human lung tissue slices or in vivo disease models. Ultimately, our findings highlight the critical necessity of utilizing complex translational pipelines to rigorously validate early screening hits before therapeutic efficacy is assumed.

## 1. Introduction

Idiopathic pulmonary fibrosis (IPF) is a chronic, progressive interstitial lung disease characterized by irreversible remodeling of the lung, progressive loss of pulmonary function, and ultimately mortality [1]. Despite major advances in disease classification, clinical management and molecular characterization of cells and states involved, therapeutic options remain limited [1,2]. Current pharmacological options are nintedanib and pirfenidone, which can slow functional decline but do not halt or reverse disease progression [3]. This unmet need continues to drive efforts to identify additional therapeutic strategies for IPF and related fibrosing lung disorders [4]. The central pathogenic feature of IPF is aberrant, uncontrolled epithelial repair coupled with the expansion and activation of fibroblast populations that deposit extracellular matrix and remodel the alveolar niche [2]. More recent single-cell, lineage-tracing studies have refined this view by showing that pulmonary fibroblasts are not a uniform population, but rather a heterogeneous and plastic population containing distinct injury-responsive states, including profibrotic *CTHRC1^+^* fibroblasts and *Scube2^+^* alveolar fibroblast populations that support epithelial homeostasis [5,6,7,8]. These findings have strengthened the rationale for targeting fibroblast state transitions rather than considering all fibroblasts as functionally equivalent. Within this framework, the balance between myofibroblast-like and lipofibroblast-like phenotypes has attracted increasing interest. Myofibroblasts are major effectors of matrix deposition and tissue stiffening [1,7,9,10], whereas lipofibroblast-associated states are linked to alveolar epithelial support, lipid handling, and repair-promoting niche functions [11,12]. Recent evidence suggests that this axis retains a degree of reversibility and may influence the capacity of fibroblasts to support epithelial regeneration [1,2]. However, marker changes and lipid accumulation in fibroblast monocultures do not by themselves establish restoration of tissue function or therapeutic relevance. This distinction matters because antifibrotic discovery pipelines often begin in reductionist systems (WI-38 fibroblasts) [13] in which fibroblast activation is modeled by transforming growth factor-β (TGF-β). Such assays are practical and mechanistically informative, but they can overestimate the significance of early hits. Increasingly, intermediate-complexity platforms such as alveolar organoids and precision-cut lung slices (PCLS) are being used to bridge the gap between two-dimensional cell culture and animal studies. Alveolar organoid systems can reveal whether mesenchymal perturbations translate into altered epithelial support, whereas PCLS preserve multicellular tissue architecture and are emerging as an important ex vivo platform for fibrosis modeling and preclinical testing [14,15,16]. In addition, no single model fully recapitulates human IPF. The bleomycin mouse model remains widely used and is valuable for proof-of-principle studies, but its translational relevance is constrained by factors including its strong early inflammatory component, dependence on intervention timing, and imperfect recapitulation of chronic human disease [17]. These limitations enhance the need for a stepwise validation across complementary model systems rather than overinterpretation of a single positive fibroblast assay [18]. Drug repurposing is particularly attractive in IPF, where the need for new therapies remains high and existing compounds offer a potential shortcut to clinical trials [1,2]. We therefore reasoned that the critical question for a putative fibroblast-modulating hit is not simply whether it alters marker expression in vitro, but whether that activity persists across progressively more complex models [19]. Neuroactive signaling pathways may contribute to fibrotic lung remodeling, and pulmonary fibrosis is also known to be commonly associated with depression and anxiety [20]. Neurogenic pathways also regulate inflammation, metabolism, and fibroblast-associated profibrotic signaling outside the central nervous system [21,22]. Desipramine is a tricyclic antidepressant (TCA) primarily used to treat depression. The mechanism of action is inhibition of the reuptake of norepinephrine, increasing its free levels in the synapse [23]. Following preliminary screening, desipramine was identified as a leading pro-lipogenic compound in TGF-β-stimulated fibroblasts, with follow-up data showing reproducible effects on fibroblast morphology, intracellular lipid accumulation, and myofibroblast-associated marker expression. In the present study, we utilize desipramine as a case study to demonstrate how tissue architectural complexity, cellular heterogeneity, and drug dynamics in 3D and in vivo systems can override promising phenotypes observed in isolated 2D screens. By advancing our testing from monocultures to human precision-cut lung slices and mouse models, we provide a clearer understanding of the compound’s true translational boundaries, underscoring the value of negative validation datasets in saving resources and refining future drug-repurposing pipelines.

## 2. Materials and Methods

### 2.1. Screening Workflow

The Prestwick Chemical Library^®^ (San Diego, CA, USA) containing 1760 compounds, primarily FDA-approved drugs (5 mM stock solutions in 100% DMSO), was screened in a 384-well plate format. WI-38 cells were seeded at a density of 2000 cells per well in 50 µL of DMEM supplemented with 10% fetal bovine serum (FBS) and penicillin/streptomycin. Following 24 h of incubation under standard culture conditions, cells were serum-starved by aspirating the culture medium using a BioTek 405 plate washer (Agilent, Santa Clara, CA, USA), briefly washing with serum-free DMEM, and adding 45 µL of serum-free DMEM supplemented with penicillin/streptomycin using a Multidrop dispenser (Thermo Fisher Scientific, Waltham, MA, USA).

After an additional 24 h, recombinant human TGF-β was added using a Multidrop dispenser by dispensing 5 µL of a 10× working solution prepared in serum-free medium, yielding a final concentration of 2 ng/mL and a final assay volume of 50 µL per well. Forty-eight hours after TGF-β stimulation, the medium was removed and replaced with 70 µL of serum-free DMEM containing penicillin/streptomycin. Compounds from the Prestwick Chemical Library were then transferred from 5 mM DMSO stock plates to assay plates using a Hamilton STAR liquid handling workstation equipped with a 384-channel dispensing head, to achieve a final compound concentration of 5 µM and a final DMSO concentration of 0.1% (*v*/*v*). Metformin was included in dedicated control wells as a positive control with final conc 5 mM. Following compound addition, cells were incubated for a further forty-eight hours.

Cells were then fixed by addition of paraformaldehyde to a final concentration of 4% and incubated for 15 min at room temperature. Lipid accumulation was assessed by LipidTOX staining according to the manufacturer’s instructions, and nuclei were counterstained with Hoechst 33342. Plates were imaged on an Operetta CLS High-Content Analysis System (Revvity) operating in confocal mode using a 20× water-immersion objective (NA 1.0). Automated image analysis was performed using Harmony/Image Analysis software (Revvity), and the mean LipidTOX fluorescence intensity and total cell number per well were quantified as primary assay readouts.

### 2.2. Screening Statistical Analysis

For each experimental condition, Z-scores were calculated relative to the distribution of control wells using the mean and standard deviation of the corresponding control population. Three independent biological replicate experiments were performed, and Z-scores were calculated for both total cell number (cell viability) and LipidTOX mean fluorescence intensity in each replicate.

To identify candidate hits, the average Z-score across the three independent experiments was calculated for each compound. Statistical significance was assessed from the averaged Z-score by calculating the corresponding *p*-value. To account for multiple testing, *p*-values were adjusted using the two-stage linear step-up procedure of Benjamini, Krieger, and Yekutieli (BKY), generating false discovery rate (FDR)-adjusted q-values.

A toxicity filter was applied to exclude compounds associated with reduced cell viability. Compounds exhibiting a significantly reduced cell number relative to controls, defined as a negative average viability Z-score across replicates and a corresponding *p*-value ≤ 0.1, were excluded from further analysis. This conservative threshold was selected to minimize the inclusion of compounds whose apparent activity could be confounded by cytotoxic effects.

To ensure robustness and reproducibility, compounds exhibiting substantial variability in effect size or direction across the three independent experiments were excluded from further consideration. Specifically, terconazole, metixene, mefloquine, and fingolimod were removed from the final hit list because their responses were not consistently reproduced across biological replicates despite meeting statistical criteria based on the averaged Z-scores.

In addition, compounds displaying intrinsic autofluorescence that interfered with image-based quantification were excluded from analysis. These compounds included merbromin disodium salt, clofazimine, dithiazanine iodide, seratrodast, indomethacin, avatrombopag, sunitinib, abemaciclib, and epirubicin hydrochloride.

The remaining non-toxic and non-autofluorescent compounds were ranked and visualized according to their average Z-score and corresponding −log10(q-value). Final hit selection was performed using the averaged Z-score together with the BKY-adjusted q-values. All statistical analyses and graphical representations were generated using GraphPad Prism 11 (GraphPad Software, San Diego, CA, USA).

### 2.3. Cell Culture

WI-38 human lung fibroblasts and MLE-12 murine lung epithelial cells were maintained under standard cell culture conditions at 37 °C in a humidified atmosphere containing 5% CO_2_. WI-38 and MLE-12 cells were cultured in DMEM with GlutaMAX supplement together with 10% fetal bovine serum (FBS) and 1% penicillin/streptomycin. Prior to TGF-β stimulation, WI-38 cells were serum-starved in depleted FBS medium for 24 h. To induce a profibrotic phenotype, WI-38 fibroblasts were stimulated with transforming growth factor-β (TGF-β) at 2 ng/mL for 2 days. Desipramine was dissolved in DMSO and used at a final concentration of 10 μM unless otherwise stated. Vehicle-treated cells received the corresponding concentration of DMSO. For further experiments, cells were seeded at different densities and treated according to the experimental design described for each assay.

### 2.4. Cell Viability Assay

WI-38 fibroblasts were seeded in 96-well plates at a density of 10,000 cells/well and allowed to attach overnight. Cells were then treated with desipramine at the indicated concentrations under TGF-β stimulation (2 ng/mL). After 96 h of treatment, cell viability was determined using alamarBlue HS (Thermo Fischer Scientific, Waltham, MA, USA) according to the manufacturer’s instructions. Briefly, reagent was added directly to the culture medium and incubated for 4 h at 37 °C. Absorbance was measured at 570 nm. Background values were subtracted where appropriate, and viability was normalized to the corresponding vehicle-treated controls.

### 2.5. LipidTOX Neutral Lipid Staining

To visualize intracellular neutral lipid accumulation, WI-38 fibroblasts were cultured on 12-well plates and treated as indicated. Cells were washed with PBS and stained with HCS LipidTOX Neutral Lipid Stain following the manufacturer’s protocol. Images were acquired using a Leica Live-imaging microscope system (Leica Camera AG, Wetzlar, Germany) under identical exposure conditions for all treatment groups. Lipid-associated fluorescence was evaluated qualitatively from representative fluorescence images.

### 2.6. Alveolosphere Assay

To assess whether desipramine-induced changes in fibroblast state translated into functional effects on epithelial organoid formation, a fibroblast-supported alveolosphere assay was performed using WI-38 fibroblasts and MLE-12 epithelial cells. WI-38 fibroblasts were first cultured under standard conditions and stimulated with TGF-β to induce a profibrotic phenotype. After this priming step, fibroblasts were used as the mesenchymal support compartment in co-culture with MLE-12 cells. For alveolosphere formation, WI-38 fibroblasts and MLE-12 cells were mixed in Matrigel and seeded in 24-well plates under organoid culture conditions. Desipramine was added to the culture medium at a final concentration of 10 μM, and medium containing the respective treatment was refreshed every 48 h. Control cultures received the corresponding vehicle. Organoids were cultured for 14 days and monitored by brightfield microscopy. At the experimental endpoint, organoid formation was assessed by microscopic imaging. At 14 days, alveolosphere cultures were collected for RNA isolation and RT-qPCR analysis of mesenchymal and epithelial marker expression, including *ACTA2*, *COL1A1*, *PLIN2*, *APOE*, *Sftpc*, *Krt8*, *Ager*, and *Hopx*.

### 2.7. Human Fibrotic PCLS

Fresh human lung tissues from IPF patients used for precision-cut lung slice (PCLS) cultures were obtained from Hannover Medical School (MHH, Hannover, Germany) in compliance with ‘The code of ethics of the world medical association’ (approval by the Ethics Committee of MHH renewed on 22 April 2015; approval number 2701-2015). Briefly, lung tissue was cannulated with a flexible catheter and the selected lung segments were inflated with warm (37 °C) low melting agarose (1.5%) prepared in Dulbecco’s Modified Eagle’s Medium Nutrient Mixture F-12 Ham (DMEM-F-12) supplemented with 15 mM HEPES, 100 U/mL penicillin, and 100 μg/mL streptomycin (all from Invitrogen Life Technologies Carlsbad, CA, USA). After polymerization of the agarose solution on ice, tissue cores of a diameter of 8 mm were prepared using a sharp rotating metal tube. Subsequently, the cores were sliced into 250–300 μm-thin slices in DMEM using a Krumdieck tissue slicer (Alabama Research and Development, Munford, AL, USA). PCLS were washed three times for 30 min in DMEM-F-12 supplemented with 15 mM HEPES, 100 U/mL penicillin, and 100 μg/mL streptomycin and left in culture for 2 days. Afterwards, they were treated with different concentrations of desipramine or vehicle control for 6 days. At endpoint, slices were processed for RNA isolation and RT-qPCR analysis. Multiple sampling sites from three biological replicates were included in order to account for tissue heterogeneity within and between samples.

### 2.8. RNA Isolation and RT-qPCR

Total RNA was isolated from WI-38 fibroblasts, MLE-12 cells, or alveolosphere cultures using RNeasy Kits (Qiagen, Hilden, Germany) according to the manufacturer’s instructions. RNA concentration and purity were determined using NanoDrop (Erlangen, Germany). Complementary DNA (cDNA) was synthesized from equal amounts of total RNA using QuantiTect Reverse Transcription Kit (Qiagen, Hilden, Germany) following the manufacturer’s protocol. Quantitative PCR was performed using GoTaq^®^ qPCR SYBR^®^ Green I master mix. Gene-expression levels were analyzed for the indicated targets, including *ACTA2*, *COL1A1*, *TAGLN*, *PLIN2*, *APOE*, *Sftpc*, *Krt8*, *Ager*, *Hopx*, *Cyr61*, and *Ccn2*, depending on the experimental setup. Relative gene expression was calculated using the ΔΔCt method and normalized to the housekeeping genes human *GAPDH*, and mouse *Hprt*. Primer sequences are provided in Table 1.

### 2.9. Western Blot Analysis

WI-38 fibroblasts were harvested and lysed in ice-cold lysis buffer containing protease and phosphatase inhibitors. Protein concentration was quantified using a standard protein assay. Equal amounts of total protein were resolved by SDS–PAGE and transferred to PVDF membranes. Membranes were blocked in TBS-T containing 10% milk and incubated overnight at 4 °C with primary antibodies against α-SMA, FGF10, PPARG and GAPDH. Following washing, membranes were incubated with species-appropriate HRP-conjugated secondary antibodies for 1 h at room temperature. Signals were detected by enhanced chemiluminescence and recorded using a digital imaging system. Band intensities were quantified by densitometry and normalized to GAPDH.

### 2.10. Bleomycin-Induced Mouse Model of Pulmonary Fibrosis

Male C57BL/6J mice were used at 6.57 weeks of age. Pulmonary fibrosis was induced by intratracheal instillation of bleomycin (1.5 U/kg) on day 0. Beginning on day 7 after injury, mice were treated once daily by intraperitoneal injection with saline or desipramine hydrochloride (10 mg/kg). Injections were administered at a volume of 10 μL/g body weight and continued until day 21. Body weight and survival were monitored during the study. At day 21, mice were euthanized, and lungs were collected for functional, histological, and biochemical analyses. These included lung function testing, H&E and Masson’s trichrome staining, morphometric assessment, hydroxyproline measurement, and BALF-based immune and cytokine analysis. The animal study was conducted by GemPharmatech Co., Ltd. (Nanjing, China). https://en.gempharmatech.com/index.html (23 July 2026).

## 3. Results

### 3.1. Initial Screening Identifies Desipramine as a Leading Pro-Lipogenic Hit in TGF-β-Treated Fibroblasts

**Figure 1:**
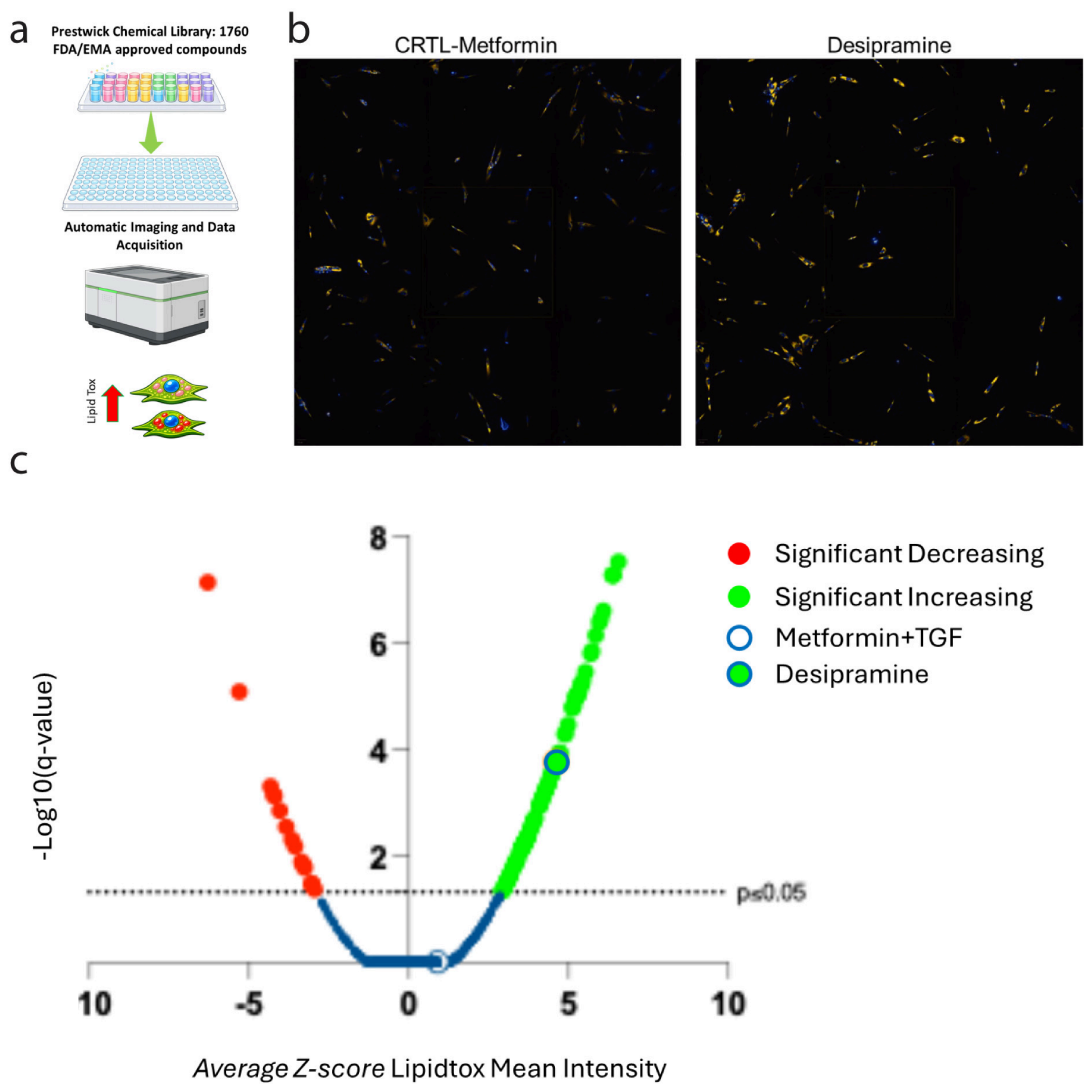
Initial screening identifies desipramine as a leading pro-lipogenic hit in TGF-β-treated fibroblasts. (**a**) Schematic overview of the primary compound-screening workflow using the Prestwick Chemical Library, comprising 1760 FDA/EMA-approved compounds, followed by automated imaging and data acquisition. Automated quantification of the lipid-associated signal (LipidTOX) served as the primary screening readout. (**b**) Representative fluorescence microscopy images of TGF-β-treated WI-38 fibroblasts stained with LipidTOX under positive control conditions (CRTL-Metformin, left) or following desipramine treatment (right). Desipramine-treated cells show a distinct increase in lipid-associated staining compared with the control condition. (**c**) Volcano plot displaying the primary screening output, mapping statistical significance (−Log10(q-value)) against effect size (Average Z-score of LipidTOX Mean Intensity). Data points represent compounds that significantly decreased (red) or increased (green) LipidTOX signal relative to the *p* ≤ 0.05 significance threshold (dashed horizontal line). The positive control setting (Metformin + TGF; open blue circle) and desipramine (green dot with blue outline) are explicitly highlighted among the top pro-lipogenic hits. Together, these data identified desipramine as a leading candidate for downstream validation.

Initial screening of an FDA-approved small-molecule library identified desipramine as one of the strongest pro-lipogenic hits in TGF-β-treated fibroblasts. This screening was carried out in collaboration with Stefano Rivetti and Luca Braga using the Prestwick Chemical Library. This was based on increased lipid-associated signal (LipidTOX) compared with typical pro-lipogenic inducer, metformin, as positive control conditions [11,24] (Figure 1). Based on the phenotypic effect observed in this primary screen and its known classification as an anti-depressive compound, desipramine was subsequently used for further validation in our fibrosis models.

### 3.2. Desipramine Is Well Tolerated at 10 μM and Was Selected for Downstream Analyses

**Figure 2:**
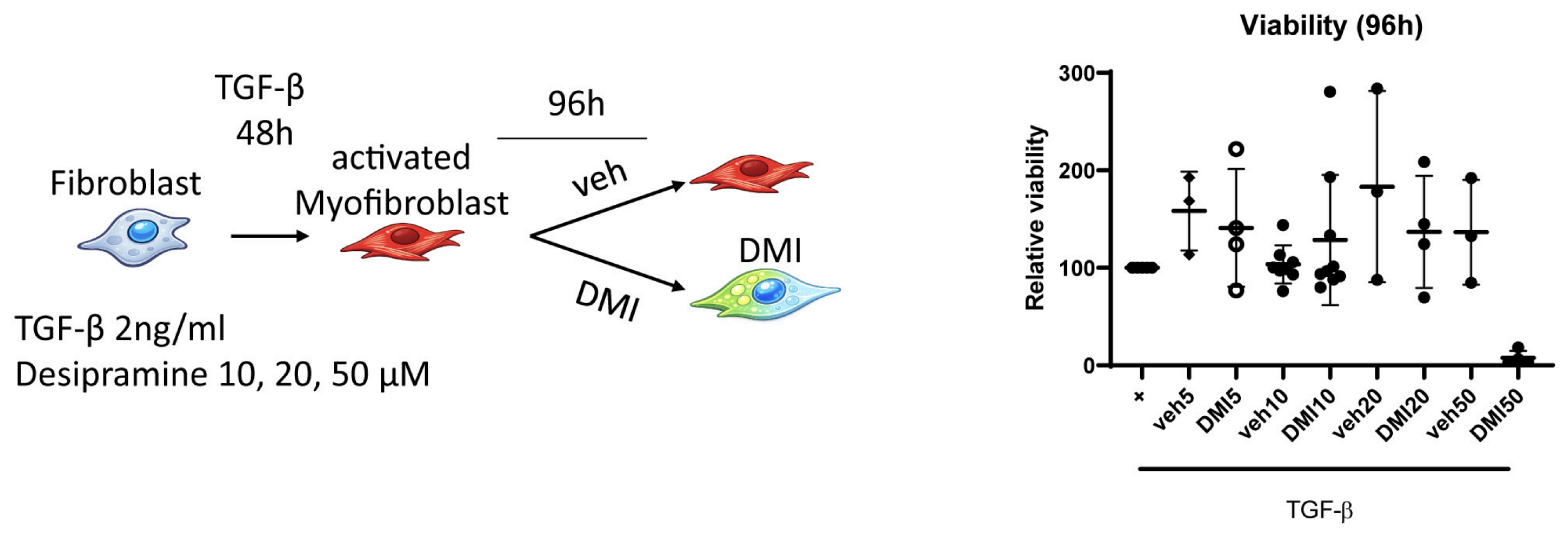
Desipramine is well tolerated at 10 μM and was selected for downstream analyses. **Left**: Experimental design: WI-38 human lung fibroblasts were stimulated with TGF-β (2 ng/mL) for 48 h to induce myofibroblast differentiation, followed by treatment with vehicle (veh) or desipramine (DMI: 10, 20, or 50 µM) for an additional 96 h. **Right**: Cell viability was assessed after 96 h of treatment using alamarBlue HS. Data are normalized to the control group and presented as mean ± SD of at least *n* = 3. Statistical significance was evaluated as non-significant using multiple independent unpaired Student’s *t*-tests, comparing each desipramine concentration directly to its respective vehicle control group. Abbreviations: TGF-β, transforming growth factor-beta; DMI, desipramine; veh, vehicle control; h, hours; SD, standard deviation.

To define a suitable working concentration for further experiments, we next assessed the effect of desipramine on WI-38 fibroblast viability under TGF-β-stimulated conditions. At 10 μM, desipramine did not significantly reduce viability after 96 h, indicating that this concentration was well tolerated in the profibrotic setting used for subsequent experiments (Figure 2). This was also consistent with the concentration used in the initial compound-library screen in which desipramine emerged as a candidate hit. In contrast, higher concentrations showed reduced cellular tolerance by microscopy, with preliminary observations indicating that 20 μM desipramine was already toxic. Therefore, 10 μM was selected as the working concentration for all subsequent experiments.

### 3.3. Desipramine Alters Fibroblast Phenotype and Marker Expression Under Profibrotic Conditions

**Figure 3:**
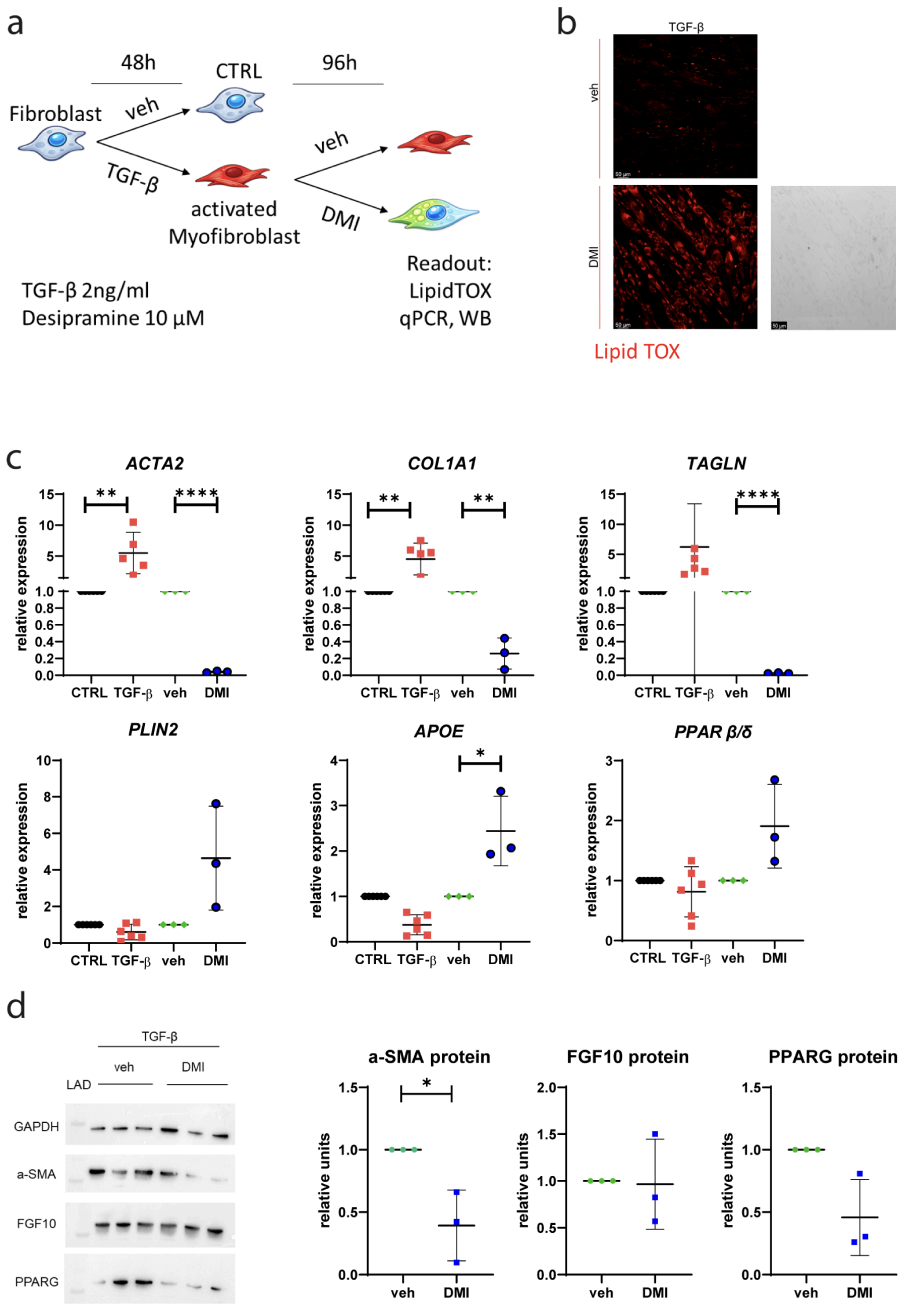
Desipramine alters fibroblast phenotype and suppresses myofibroblast-associated markers under profibrotic conditions. (**a**) Schematic overview of the experimental design. WI-38 fibroblasts were stimulated with TGF-β (2 ng/mL) for 48 h to induce myofibroblast activation and were then treated with vehicle (veh) or desipramine (DMI) (10 μM) for 96 h. Readouts included LipidTOX staining, RT-qPCR, and Western blotting. (**b**) Representative LipidTOX images show increased lipid-associated staining in TGF-β-stimulated WI-38 fibroblasts treated with desipramine compared with vehicle-treated cells. Scale bar = 50 µm. (**c**) RT-qPCR analysis of WI-38 fibroblasts under control, TGF-β, vehicle, and desipramine-treated conditions. Under TGF-β-stimulated conditions, desipramine reduced the expression of canonical profibrotic markers, including *ACTA2*, *COL1A1*, and *TAGLN*, while modulating selected lipid-associated markers such as *PLIN2* and *APOE*. *PPARβ/δ* expression showed no consistent increase under the conditions tested. Data are normalized to the control groups and presented as mean ± SD of at least *n* = 3 independent biological experiments. Statistical significance was evaluated using an unpaired Student’s *t*-test for comparisons between the desipramine-treated group and its respective vehicle control under TGF-β conditions. Significance symbols: * *p* < 0.05, ** *p* < 0.01, **** *p* < 0.0001. (**d**) Representative Western blots and densitometric quantification show a significant reduction in α-SMA protein following desipramine treatment, whereas FGF10 and PPARG protein levels remained variable and were not consistently increased. Data are normalized to the control groups and presented as mean ± SD of *n* = 3 independent biological experiments. Statistical significance was evaluated using an unpaired Student’s *t*-test for comparisons between the desipramine-treated group and its respective vehicle control under TGF-β conditions. Significance symbols: * *p* < 0.05. Abbreviations: TGF-β, transforming growth factor-beta; DMI, desipramine; veh, vehicle control; h, hours.

Having already defined 10 μM as the well tolerated concentration, we next examined whether desipramine at this dose could affect fibroblast phenotype under profibrotic conditions. In line with the initial screening results, desipramine treatment altered fibroblast morphology and increased lipid-associated signal, particularly in TGF-β-treated cells, suggesting an effect on fibroblast state (Figure 3b). We then assessed whether these phenotypic alterations were accompanied by changes in myofibroblast- and lipogenic-associated markers. At the mRNA level, desipramine reduced the expression of canonical profibrotic markers under profibrotic conditions, including *ACTA2*, *COL1A1* and *TAGLN*, while modulating selected lipogenic-associated markers such as *PLIN2* and *APOE* (Figure 3c). Under naïve physiological conditions, desipramine showed a modest to no effect on these markers (Figure S1). At the protein level, desipramine significantly reduced α-SMA expression, consistent with attenuation of the myofibroblast phenotype. In contrast, FGF10 and PPARG protein levels showed greater variability and no clear increase under the conditions tested (Figure 3d). Together, these findings indicate that desipramine exerts an effect on fibroblast phenotype, with the most robust evidence seen for suppression of the myofibroblast-associated program.

### 3.4. Desipramine-Treated Fibroblasts Partially Improve Alveolosphere Formation

**Figure 4:**
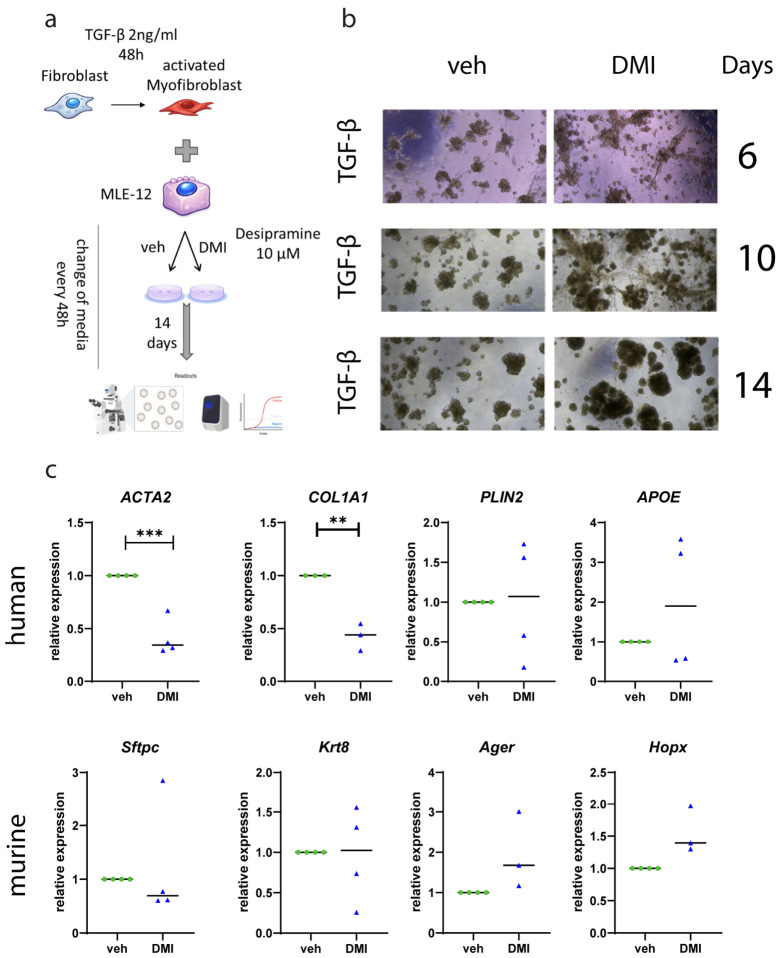
Desipramine-treated fibroblasts partially improve alveolosphere formation in a WI-38/MLE-12 co-culture model. (**a**) Schematic overview of the alveolosphere assay. WI-38 fibroblasts were first stimulated with TGF-β (2 ng/mL) for 48 h to induce a profibrotic state and were then co-cultured with MLE-12 epithelial cells in Matrigel. Cultures were treated with vehicle (veh) or desipramine (DMI) (10 μM), with media changed every 48 h, and organoids were analyzed after 14 days. (**b**) Representative brightfield images of fibroblast-supported alveolospheres at days 6, 10, and 14. Desipramine-treated cultures showed improved organoid growth and morphology compared with vehicle-treated profibrotic controls. (**c**) RT-qPCR analysis of alveolosphere cultures. Human mesenchymal markers (*ACTA2*, *COL1A1*, *PLIN2*, *APOE*) and murine epithelial markers (*Sftpc*, *Krt8*, *Ager*, *Hopx*) were assessed after 14 days. Desipramine significantly reduced *ACTA2* and *COL1A1* expression, whereas lipogenic-associated and epithelial markers showed more variable responses. Data are normalized to the vehicle group and presented as mean ± SD of *n* at least 3. Statistical analysis was performed using unpaired Student’s *t*-tests to evaluate differences between each specific treatment condition and its respective control. Significance symbols: ** *p* < 0.01, *** *p* < 0.005. Abbreviations: TGF-β, transforming growth factor-beta; DMI, desipramine; veh, vehicle control; h, hours; RT-qPCR, reverse transcription-quantitative polymerase chain reaction.

To determine whether the desipramine-induced changes observed in fibroblasts translate into functional effects on epithelial growth, we next used a fibroblast-supported alveolosphere model composed of WI-38 cells and MLE-12 epithelial cells. WI-38 fibroblasts were first challenged toward a profibrotic state by TGF-β treatment and were subsequently co-cultured with MLE-12 cells in the alveolosphere assay, with desipramine added in the cell culture medium (Figure 4a). Microscopic evaluation showed that desipramine treatment resulted in qualitative morphological variations, characterized by changes in aggregate appearance and cluster distribution under TGF-β pressure. Because a non-profibrotic baseline control was not evaluated, these morphological changes do not imply a restoration toward a healthy baseline state (Figure 4b). To further examine this effect at the molecular level, we analyzed mesenchymal and epithelial marker expression in the alveolosphere cultures by RT-qPCR. Desipramine significantly reduced expression of the myofibroblast-associated markers *ACTA2* and *COL1A1*, consistent with attenuation of the profibrotic mesenchymal program seen in cell culture level. Lipogenic-associated markers such as *PLIN2* and *APOE* displayed greater variability across replicates. Analysis of epithelial markers revealed no uniform increase across all readouts. While markers such as *Krt8*, *Ager*, and *Hopx* were maintained or modestly altered, *Sftpc* did not show a clear increase under desipramine treatment in this setting (Figure 4c). Taken together, these data suggest that desipramine alters organoid formation primarily through partial modulation of the fibroblast state, while the epithelial transcriptional response remains limited or heterogeneous.

### 3.5. Direct Desipramine Treatment Does Not Elicit a Consistent Epithelial Transcriptional Response in MLE-12 Cells

Next, to determine whether desipramine directly affects epithelial cell state, we treated MLE-12 cells under the same experimental framework and analyzed epithelial and mechanosensitive marker expression by RT-qPCR. In contrast to the fibroblast and alveolosphere settings, desipramine treatment on TGF-β-induced epithelium did not cause a consistent transcriptional response in MLE-12 cells. Expression of epithelial markers, including *Sftpc*, *Krt8*, *Ager*, and *Hopx*, remained variable across replicates and did not show a uniform increase under desipramine treatment. Similarly, the mechanosensitive genes *Cyr61* and *Ccn2* were not consistently altered (Figure S2). These findings suggest that the beneficial effect observed in the alveolosphere model is unlikely to be mediated by a strong cell-autonomous effect of desipramine on epithelial cells and is more consistent with a context-dependent effect involving mesenchymal support.

### 3.6. Desipramine Shows Variable Effects in Human Fibrotic Precision-Cut Lung Slices

**Figure 5:**
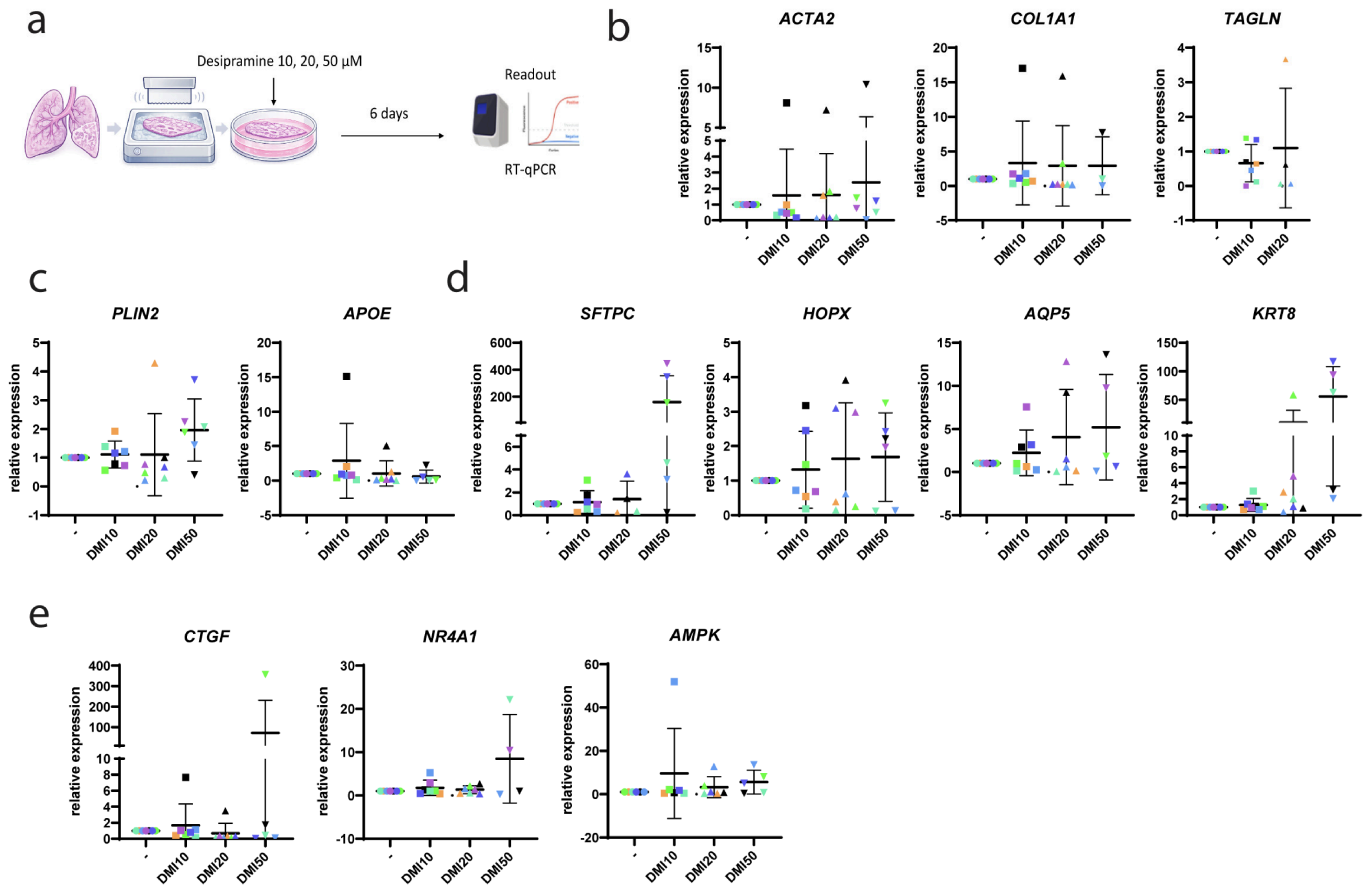
Desipramine shows variable effects in human fibrotic precision-cut lung slices. (**a**) Schematic workflow of the ex vivo human PCLS model. Slices obtained from different lung regions of human patients were cultured and treated with increasing concentrations of desipramine (DMI; 10, 20, or 50 µM) or vehicle control (-) for 6 days prior to RT-qPCR analysis. (**b**–**e**) Relative mRNA expression levels of (**b**) mesenchymal markers (*ACTA2*, *COL1A1*, *TAGLN*), (**c**) lipid-associated markers (*PLIN2*, *APOE*), (**d**) alveolar epithelial markers (*SFTPC*, *HOPX*, *AQP5*, *KRT8*), and (**e**) downstream signaling targets (*CTGF*, *NR4A1*, *AMPK*). Individual data points are color- and shape-coded to represent the at least 3 distinct precision-cut lung slices which were derived from multiple distinct anatomical regions across N = 3 independent biological patient donors (two patients yielding 2 regional slices each, and one patient yielding 3 regional slices). Due to the inherent heterogeneity of primary human tissue and varying basal expression levels, some target transcripts fell below the limit of detection in specific samples. Therefore, data represent a minimum of *n* = 3 slices. Data are presented as mean ± SD and are interpreted strictly as exploratory and descriptive due to the limited biological donor number. Exploratory statistical analysis was conducted using multiple independent unpaired Student’s *t*-tests to compare each desipramine concentration directly to vehicle control group. None of comparisons reached statistical significance. Abbreviations: PCLS, precision-cut lung slices; DMI, desipramine; RT-qPCR, reverse transcription-quantitative polymerase chain reaction; SD, standard deviation.

Although desipramine improved organoid formation in the WI-38/MLE-12 alveolosphere model, we next asked whether this effect would persist in a more complex human tissue context. We evaluated the effects of desipramine in a highly complex human ex vivo model utilizing primary human precision-cut lung slices (PCLS) from N = 3 independent donors. Exploratory statistical analysis via unpaired *t*-tests revealed that desipramine treatment did not result in statistically significant changes in the expression of canonical profibrotic, lipid-associated, epithelial and additional response markers compared to vehicle controls. Instead, the human PCLS responses were characterized by high intra-donor and spatial variability, demonstrating only descriptive, non-significant baseline trends. These findings underscore the critical translational boundaries of the compound when transitioning from simplified 2D cell cultures to heterogeneous human lung tissue (Figure 5).

### 3.7. Desipramine Did Not Show a Robust Antifibrotic Effect in the Bleomycin Mouse Model

**Figure 6:**
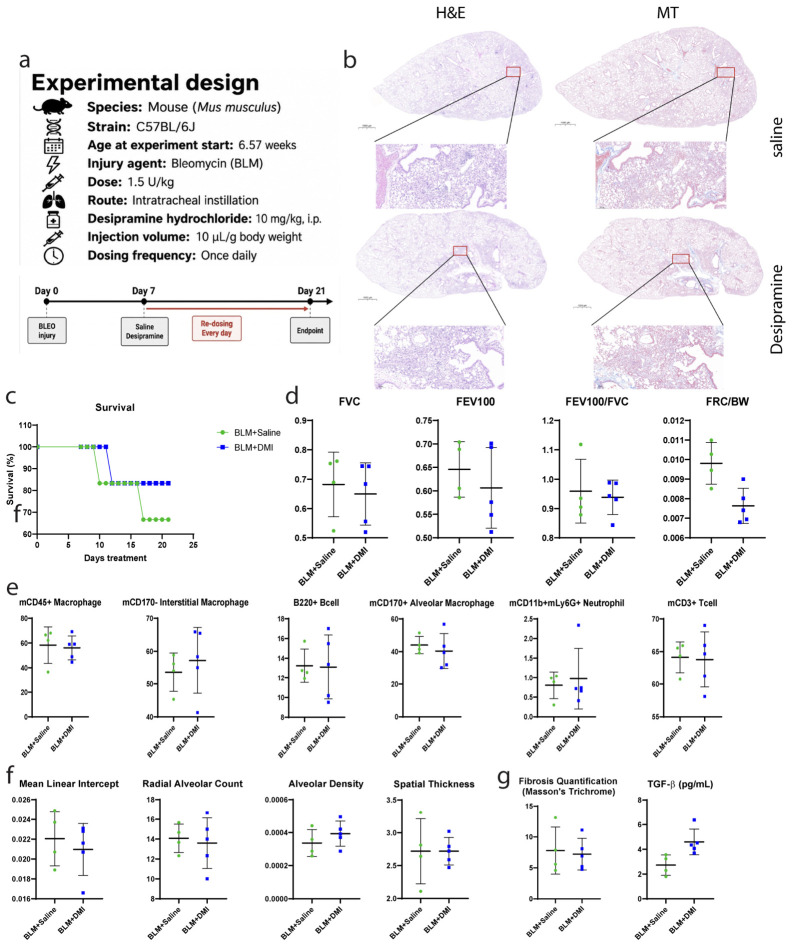
Desipramine in the bleomycin mouse model of pulmonary fibrosis. (**a**) Experimental design. C57BL/6J mice received intratracheal bleomycin (1.5 U/kg) on day 0 to induce pulmonary fibrosis. Beginning on day 7, mice were treated once daily by intraperitoneal injection with saline or desipramine hydrochloride (10 mg/kg) until day 21. (**b**) Representative hematoxylin and eosin (H&E) and Masson’s trichrome (MT) images of bleomycin-injured lungs from saline- and desipramine-treated mice, with corresponding higher-magnification insets. (**c**) Endpoint analyses including (**c**) survival, (**d**) lung function parameters (FVC, FEV100, FEV100/FVC, and FRC/BW), (**e**) BALF immune-cell populations (mCD45+ macrophages, mCD170− interstitial macrophages, mCD170+ alveolar macrophages, mCD11b + Ly6G + neutrophils, B220+ B cells, and mCD3+ T cells), (**f**) morphometric parameters (mean linear intercept, radial alveolar count, alveolar density, and septal thickness), (**g**) histological fibrosis quantification from Masson’s trichrome staining, and soluble TGF-β levels. Desipramine was generally well tolerated but did not produce a clear and consistent improvement across functional, inflammatory, morphometric, or histological fibrosis endpoints compared with bleomycin–saline controls. Data are presented as mean ± SD of BLM (bleomycin) + saline *n* = 4 and BLM + DMI (desipramine) *n* = 5 mice. Statistical significance was evaluated using a Student’s unpaired *t*-test. Desipramine was generally well tolerated but did not produce a clear and consistent improvement across functional, inflammatory, morphometric, or histological fibrosis endpoints compared with bleomycin–saline controls. Abbreviations: FVC, forced vital capacity; FEV100, forced expiratory volume in 100 ms; FRC/BW, functional residual capacity normalized to body weight; BALF, bronchoalveolar lavage fluid; H&E, hematoxylin and eosin; MT, Masson’s trichrome; i.p., intraperitoneal; SD, standard deviation.

Because the PCLS data indicated heterogeneous responses in human tissue, we next asked whether desipramine would show better efficacy in an in vivo model of established fibrosis. To further assess the translational relevance of desipramine, we evaluated its potential effect in a more complex, bleomycin-induced mouse model of pulmonary fibrosis. Pulmonary injury was induced in C57BL/6J mice by intratracheal bleomycin administration (1.5 U/kg) on day 0, and therapeutic treatment was initiated on day 7, when animals received daily intraperitoneal injections of desipramine hydrochloride (10 mg/kg) until the endpoint on day 21 (Figure 6a). Under these conditions, desipramine was well tolerated in vivo, as evidenced by its minimal impact on survival compared to saline condition (Figure 6c). We next assessed whether desipramine improved physiological or fibrotic endpoints. Lung function measurements, including FVC and FEV100, did not show a clear improvement in the desipramine-treated group compared with bleomycin–saline control (Figure 6d). Similarly, hydroxyproline content and histological fibrosis readouts did not indicate a robust reduction in fibrotic burden. Quantification of Masson’s trichrome-positive tissue showed at most a modest decrease in the desipramine group, while H&E-based morphometric parameters, including mean linear intercept, radial alveolar count, alveolar density, and septal thickness, remained comparable across groups (Figure 6f,g). Analysis of BALF immune cell populations and soluble mediators also revealed only limited changes (Figure 6e). Together, these findings indicate that desipramine did not produce a strong and consistent antifibrotic effect in vivo.

## 4. Discussion

In this study, while desipramine showed clear biological activity in reductionist fibroblast assays, this activity did not translate into a robust antifibrotic signal in higher-complexity systems. In WI-38 human fibroblasts, desipramine was selected at 10 μM as the working concentration because viability was preserved at that dose under TGF-β-stimulated conditions. Higher exposure was less well tolerated and 20 μM appeared toxic by microscopy. Under these conditions, desipramine altered fibroblast morphology, increased lipid-associated staining, reduced myofibroblast-associated readouts, and lowered α-SMA protein. In the alveolosphere assay, desipramine improved organoid formation and reduced *ACTA2*, but epithelial marker responses were variable. In human fibrotic PCLS, responses were heterogeneous, and in the bleomycin model even though desipramine was tolerated well, it did not produce clear improvement in lung function, hydroxyproline, or overall histological fibrosis burden. Taken together, our data support desipramine as a context-dependent modulator of fibroblast state rather than a strong antifibrotic lead. This pattern is biologically plausible in light of the current understanding of fibroblast plasticity. Recent evidence supports a reversible axis between lipofibroblast-like and activated myofibroblast states, and WI-38 cells have been proposed as a model for studying that switch [13]. In parallel, alveolar lipofibroblasts are increasingly recognized as a niche component that supports epithelial homeostasis and repair [6,10,12]. Against that, the combination of lipid accumulation, morphological change, and suppression of *ACTA2/COL1A1* in our fibroblast experiments indicates only a partial shift away from a profibrotic state. However, the literature also argues that marker changes in fibroblasts are not equivalent to restoration of tissue-level function. That distinction is important here, because our data suggest attenuation of the myofibroblast program without convincing evidence of a stable, fully functional lipofibroblast-like conversion. The protein data particularly support that interpretation. In our hands, α-SMA was the strongest and most reproducible protein-level readout affected by desipramine, whereas PPARG and FGF10 were not consistently increased at the protein level. This indicates that desipramine acts as a context-dependent modulator of fibroblast states rather than driving a true, complete lipofibroblast state modulation. More precisely, we suggest that desipramine dampens selected profibrotic features while producing an incomplete or unstable pro-lipogenic program. According to the literature, desipramine acts as a functional inhibitor of acid sphingomyelinase (ASM) and behaves as a lysosomotropic compound. Desipramine can accumulate in acidic compartments, functionally inhibit ASM, and disturb lysosomal lipid handling in fibroblasts [22,25]. Therefore, at least part of the lipid phenotype we observed may reflect lysosomal drug biology or altered lipid trafficking rather than lineage phenotypic shift [26]. Because we did not directly measure intracellular ASM activity or lysosomal cargo transport in this study, further mechanistic work is required to definitively separate true lipofibroblast phenotypic shift from non-specific lysosomal lipid retention. The alveolosphere data nonetheless suggest that desipramine is not acting as a toxic factor. In our WI-38/MLE-12 co-culture model, desipramine improved organoid formation and reduced *ACTA2* and *COL1A1*, so we suggest that desipramine-induced pro-lipogenic phenotypic and marker alterations can have functional consequences for epithelial growth [2,13]. At the same time, direct treatment of MLE-12 cells did not elicit a strong, consistent epithelial transcriptional response. This suggests that the organoid benefit is more likely mediated through the mesenchymal compartment than through a dominant epithelial cell-autonomous effect. That interpretation is also consistent with prior work showing that lipid-droplet-bearing fibroblast states support alveolosphere formation, whereas activated myofibroblast states are less supportive [13]. The limitation is that our epithelial qPCR panel was heterogeneous, so the functional organoid improvement should be interpreted as context-dependent rather than as full epithelial rescue. A key question is why these encouraging fibroblast and organoid findings weakened in complex systems such as PCLS and in vivo. One explanation is the model complexity itself. PCLS preserve native architecture and multicellular interactions and are increasingly viewed as an important translational bridge between cell culture and animal studies. At the same time, they are heterogeneous regarding structure, cell composition, baseline injury, and donor-specific disease state [14,27]. The marked variability in our fibrotic PCLS experiments is therefore not surprising and should not be dismissed as technical failure. Instead, it likely indicates that the desipramine-responsive state seen in WI-38 cells is not uniformly present or accessible across intact human fibrotic tissue. According to the literature, the same logic extends to the bleomycin model. The bleomycin model remains useful, but its inflammatory kinetics, timing dependence, and imperfect overlap with chronic human fibrosis are well recognized [17,18]. The lack of measurable antifibrotic efficacy or physiological recovery in vivo, despite the compound being well tolerated at 10 mg/kg, aligned with the literature baseline, points toward a critical pharmacokinetic and tissue-compartment gap. Although the administration used in our work represents a validated preclinical systemic threshold shown to effectively alter peripheral inflammatory pathways and physiological kinetics in other rodent models [22,28,29], it does not guarantee that localized drug levels within the deep pulmonary parenchyma reach the exact exposure window required to interrupt the profibrotic fibroblast state observed in vitro. Furthermore, we did not perform direct pharmacokinetic profiling to verify local target engagement or compound concentration within the deep lung parenchyma. Desipramine is a lipophilic compound historically optimized for central nervous system distribution. Consequently, fast peripheral clearance or insufficient tissue penetration might have limited its local availability in the lung. This highlights the definitive need for future work to implement precise dose-exposure matching across models. Evaluating alternative delivery routes, such as localized intratracheal aerosolization, or testing a broader dose–response will be required to establish exposure–response relationships and evaluate the true therapeutic boundaries of the compound in complex tissue architectures.

In our experimental design, in order to approach therapeutically, dosing began on day 7 and continued to day 21. A critical variable in the bleomycin model is the timing of drug administration, which dictates whether a compound is evaluated for prophylactic or therapeutic efficacy [18]. According to studies, the first 5 days following intratracheal bleomycin instillation are heavily dominated by acute epithelial damage, severe alveolar inflammation, and an initial cytokine storm [17]. In our experimental model, by treating at day 7, we allowed this early inflammatory phase to settle, establishing a proper therapeutic testing window. This timeline ensures that desipramine was challenged to modulate or potentially reverse an active, ongoing fibrotic program, rather than simply acting as an early anti-inflammatory or cytoprotective agent. While this treatment regimen represents a translational benchmark often utilized to evaluate antifibrotic leads [24], the lack of measurable functional or histological improvement in our hands suggests that dampening the selected fibroblast traits in vitro was insufficient to arrest cell- and tissue-level remodeling once the chronic fibrotic one was fully established.

Our findings provide a crucial cautionary example against relying solely on reductionist 2D screening pipelines for drug repurposing in IPF. While desipramine exhibited initial antifibrotic potential in standard 2D fibroblast cultures, these effects completely failed to translate to more complex biological systems. Furthermore, our validation efforts highlighted the high inherent variability of human PCLS, which underscores the difficulty of translating in vitro hits into heterogeneous human tissue. The most meaningful conclusion is not that desipramine is ineffective in every fibrotic context, nor that the fibroblast findings were artifactual, but that fibroblast-state modulation alone is not enough to nominate a repurposed drug as a therapeutic lead. For future work, the most informative next steps would be to direct target-engagement studies in PCLS, dose–exposure matching across models, and mechanistic separation of lipofibroblast-supportive phenotype shift from lysosomal lipid accumulation. In conclusion, the present study is valuable as a translational filtering study: it identifies where the desipramine signal is real, where it weakens, and why early fibroblast phenotypes should not be overinterpreted as evidence of antifibrotic efficacy.

### Study Limitations

While our multi-tier validation pipeline offers a look at the translational boundaries of desipramine, certain technical limitations should be noted. First, our structural analysis of the 3D alveolosphere co-cultures relied on high-resolution qualitative imaging rather than automated morphometric quantification. The nature of these complex co-cultures is characterized by irregular multi-lobular growth, massive clustering, and the development of optically dense core regions, hindered automated segmentation software and manual counting. We countered this by pairing our morphological observations with sensitive, compartment-specific RT-qPCR. Because changes at the RNA level do not always translate directly to altered protein abundance and given that no significant transcriptional shifts were detected within the epithelial compartment, prioritizing this sensitive transcriptomic approach allowed us to cleanly assess early compartment-specific responses before pursuing resource-heavy protein assays.

Furthermore, expanding our Western blot or immunofluorescence validation to the human PCLS and in vivo models presented severe logistical and ethical constraints. Procuring the high volume of human donor tissue necessary to achieve adequate statistical power for large-scale protein quantification in PCLS is frequently unfeasible. Similarly, for the in vivo part, expanding the animal cohorts solely for extensive tissue-level protein validation was deemed ethically unjustifiable, particularly given the absence of a strong primary phenotypic or functional therapeutic effect. Finally, our in vivo modeling was restricted to a single systemic dose (10 mg/kg i.p.) selected based on the established literature. While evaluating a broader range of concentrations or exploring localized delivery routes, such as inhalation to maximize parenchymal target engagement, while limiting systemic clearance and neuroactive side effects would be highly valuable, the lack of an initial efficacy limited further dose-escalation studies.

It is critical to note that the biological alterations observed in our 3D alveolospheres, human PCLS, and in vivo mouse models are restricted to bulk transcriptomic changes and qualitative imaging profiles. Because downstream protein expression was not validated in these high-complexity environments, these changes must not be interpreted as evidence of stable lipofibroblast lineage conversion or functional fibroblast reprogramming. Instead, desipramine appears to induce context-dependent, superficial transcript alterations in reductionist models that completely fail to manifest as robust phenotypic shifts in multi-cellular human tissues or functional protection in vivo.

Recognizing these boundaries prevents the over-interpretation of our screening data while highlighting where future target-engagement strategies should be refined.

## 5. Future Perspectives

The present findings indicate that desipramine may still be useful as a probe compound for studying fibroblast plasticity, even if it does not qualify as a strong antifibrotic lead in its current form. Future work should focus on separating ASM/lysosomal effects from bona fide mesenchymal reprogramming, defining exposure–response relationships across fibroblasts, PCLS, and in vivo tissue, and identifying the cellular contexts in which desipramine-responsive states are present. To circumvent the localized pharmacokinetic and tissue-compartment gaps observed here, future strategies should explore targeted pulmonary delivery modalities, such as inhalation, to optimize parenchymal bioavailability while limiting systemic clearance. It would also be relevant to test whether desipramine performs better in combination settings, particularly in models where oxidative stress, macrophage activation, or sphingolipid signaling are dominant drivers of pathology. More generally, this study highlights the need for antifibrotic screening strategies that move beyond marker suppression in fibroblasts and incorporate functional and tissue-level validation at an earlier stage.

## Figures and Tables

**Table 1:** List of primers used in this study.

Primer	Sequence
*hGAPDH fw.*	ACCCAGAAGACTGTGGATGG
*hGAPDH rev.*	GTGTCGCTGTTGAAGTCAGAG
*hACTA2 fw.*	CTGTTCCAGCCATCCTTCAT
*hACTA2 rev.*	TCATGATGCTGTTGTAGGTGG
*hCOL1A1 fw.*	ATGTTCAGCTTTGTGGACCTC
*hCOL1A1 rev.*	CTGTACGCAGGTCATTGGTG
*hTAGLN fw.*	CCGTGGAGATCCCAACTGG
*hTAGLN rev.*	CCATCTGAAGGCCAATGACAT
*hPLIN2 fw.*	TCAGCTCCATTCTACTGTTCACC
*hPLIN2 rev.*	CCTGAATTTTCTGATTGGCA
*hAPOE fw.*	GTTGCTGGTCACATTCCTGG
*hAPOE rev.*	GCAGGTAATCCCAAAAGCGAC
*hSFTPC fw.*	AGTGCCTACGCTTAAGCTG
*hSFTPC rev.*	CGTGGGGGCTCTTTTCC
*hKRT8 fw.*	ACAAGGTAGAGCTGGAGTCTCG
*hKRT8 rev.*	AGCACCACAGATGTGTCCGAGA
*HOPX_h_For*	ATTCCACCACGCTGTGCCTCAT
*HOPX_h_Rev*	AGTCTGTGACGGATCTGCACTC
*AQP5_h_For*	GCCACCTTGTCGGAATCTACT
*AQP5_h_Rev*	GGCTCATACGTGCCTTTGATG
*hCTGF_F*	CAGGCTAGAGAAGCAGAGC
*hCTGF_R*	TGGAGATTTTGGGAGTACGG
*hNR4A1fw*	ATGCCCTGTATCCAAGCCC
*hNR4A1rv*	GTGTAGCCGTCCATGAAGGT
*hAMPKfw*	AGGAAGAATCCTGTGACAAGCAC
*hAMPKrv*	CCGATCTCTGTGGAGTAGCAGT
*mHprt fw.*	CCTAAGATGAGCGCAAGTTGAA
*mHprt rev.*	CCACAGGACTAGAACACCTGCTAA
*mSftpc F*	GGTCCTGATGGAGAGTCCAC
*mSftpc R*	GATGAGAAGGCGTTTGAGGT
*mHopx F*	ACCACGCTGTGCCTCATC
*mHopx R*	GCGCTGCTTAAACCATTTCT
*mAger F*	GCCACTGGAATTGTCGATGAGG
*mAger R*	GCTGTGAGTTCAGAGGCAGGAT
*mCcn2_F*	GGCCTCTTCTGCGATTTCG
*mCcn2_R*	GCAGCTTGACCCTTCTCGG
*mCyr61_F*	CTCCAGAATCTACCAAAACGGG
*mCyr61_R*	CGTCCAGGGAGTCCTTAATGC

## Data Availability

The data presented in this study are available in the article and its Supplementary Materials.

## Supplementary Materials

The following supporting information can be downloaded at: https://www.mdpi.com/article/10.3390/cells15151344/s1, Figure S1: Desipramine has limited effects on marker expression in naïve WI-38 fibroblasts; Figure S2: Direct desipramine treatment does not elicit a consistent epithelial transcriptional response in MLE-12 cells.

## Abbreviations

The following abbreviations are used in this manuscript:

ACTA2	Actin Alpha 2, Smooth Muscle
AGER	Advanced Glycosylation End-Product Specific Receptor
alamarBlue HS	alamarBlue High Sensitivity
α-SMA	Alpha-Smooth Muscle Actin
APOE	Apolipoprotein E
ASM	Acid Sphingomyelinase
BALF	Bronchoalveolar Lavage Fluid
BLM	Bleomycin
CCN2	Cellular Communication Network Factor 2
cDNA	Complementary DNA
COL1A1	Collagen Type I Alpha 1 Chain
CTGF	Connective Tissue Growth Factor
CTHRC1	Collagen Triple Helix Repeat Containing 1
Cyr61	Cysteine-Rich Angiogenic Inducer 61
DMEM	Dulbecco’s Modified Eagle Medium
DMSO	Dimethyl Sulfoxide
ECM	Extracellular Matrix
FBS	Fetal Bovine Serum
FGF10	Fibroblast Growth Factor 10
FEV100	Forced Expiratory Volume in 100 ms
FRC/BW	Functional Residual Capacity/Body Weight
FVC	Forced Vital Capacity
GAPDH	Glyceraldehyde-3-Phosphate Dehydrogenase
H&E	Hematoxylin and Eosin
Hopx/HOPX	Homeodomain-Only Protein Homeobox
HRP	Horseradish Peroxidase
Hprt	Hypoxanthine Phosphoribosyltransferase
i.p.	Intraperitoneal
IPF	Idiopathic Pulmonary Fibrosis
KRT8/Krt8	Keratin 8
NR4A1	Nuclear Receptor Subfamily 4 Group A Member 1
PCLS	Precision-Cut Lung Slices
PLIN2	Perilipin 2
PPARG	Peroxisome Proliferator-Activated Receptor Gamma
Scube2	Signal Peptide, CUB Domain and EGF Like Domain Containing 2
SFTPC/Sftpc	Surfactant Protein C
TAGLN	Transgelin
TBS-T	Tris-Buffered Saline with Tween 20
TCA	Tricyclic Antidepressant
TGF-β	Transforming Growth Factor Beta
