# Supplementary material for: Stepwise Translational Validation of the Screening Hit Desipramine Reveals Limits of Fibroblast-State Modulation in Lung Fibrosis
Source: Cells. 2026 Jul 27;15(15):1344. doi: 10.3390/cells15151344 (PMC13465335; doi:10.3390/cells15151344)
Supplement: Supplementary file 1 [file cells-15-01344-s001.zip › cells-4431096-supplementary.pdf]

Supplementary Figure S1. Desipramine has limited effects on marker expression in naïve WI-38 fibroblasts.

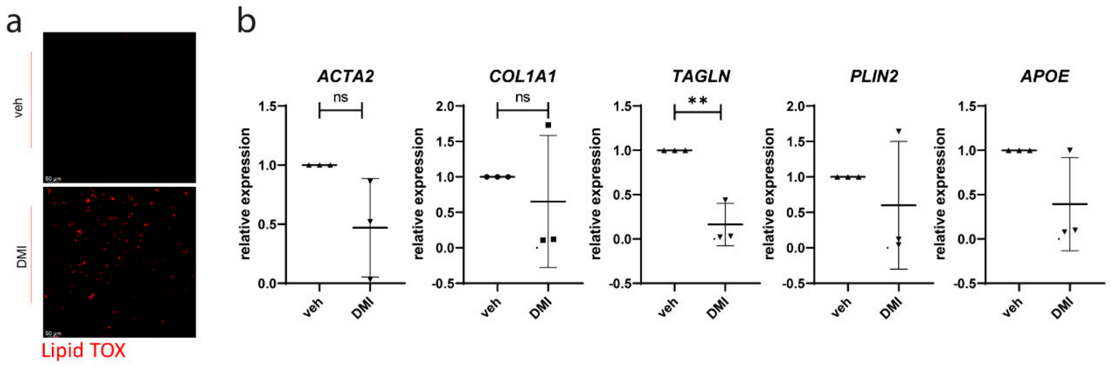

Supplementary Figure S1. Desipramine has limited effects on marker expression in naïve WI-38 fibroblasts. Naïve WI-38 human lung fibroblasts were treated with vehicle control (veh) or desipramine (DMI; 10 μM) under baseline, non-stimulated conditions for 96 h and analyzed via LipidTOX staining and RT-qPCR. (a) Representative images showing LipidTOX neutral lipid staining in vehicle- or desipramine-treated naïve WI-38 fibroblasts. Scale bar = 50 μm. (b) RT-qPCR expression analysis of the profibrotic markers (*ACTA2, COL1A1, TAGLN*) and lipid-associated markers (*PLIN2, APOE*). In the absence of profibrotic TGF-β stimulation, desipramine produced only modest transcriptional effects, with a minor trend toward reduced *ACTA2* and a significant reduction in *TAGLN*, whereas *COL1A1, PLIN2,* and *APOE* remained unaltered. Data are normalized to the vehicle control group and presented as mean ± SD of n = 3. Statistical analysis was performed using an unpaired Student's t-test comparing desipramine directly to the vehicle control. Significance symbols: *$p < 0.05$, and n.s. = not significant. Abbreviations: DMI, desipramine; veh, vehicle control; RT-qPCR, reverse transcription-quantitative polymerase chain reaction; SD, standard deviation; h, hours.

Supplementary Figure S2. Direct desipramine treatment does not elicit a consistent epithelial transcriptional response in MLE-12 cells

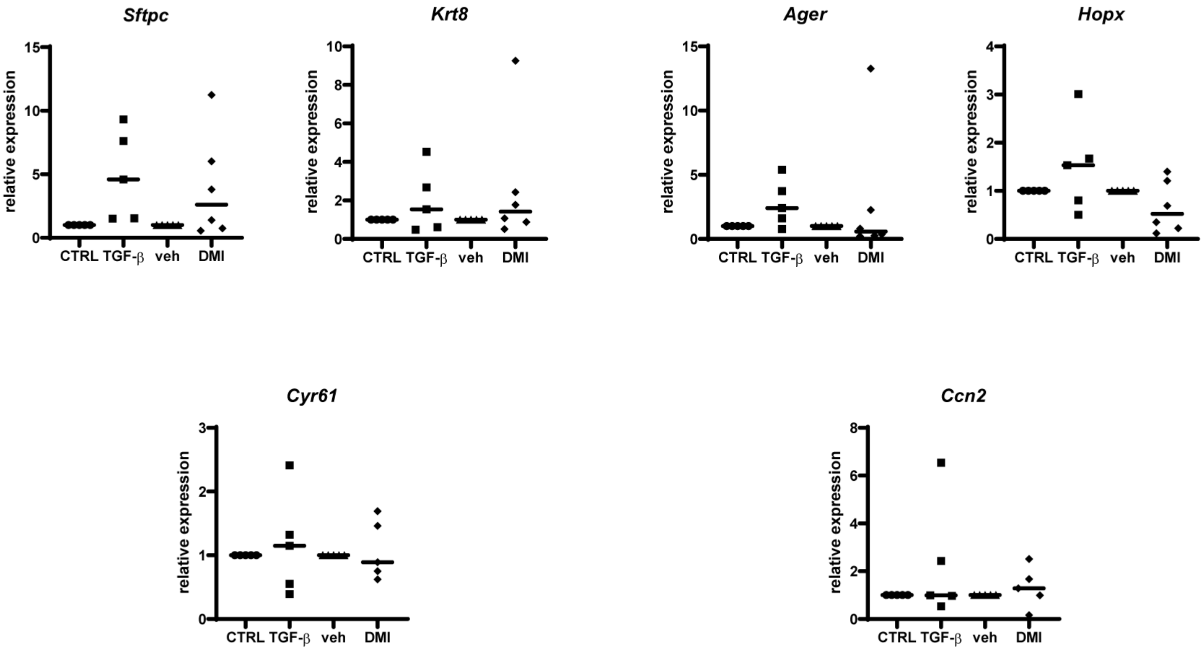

Supplementary Figure S2. Direct desipramine treatment does not elicit a consistent transcriptional response in MLE-12 epithelial cells. To assess whether desipramine exerts a direct effect on epithelial cell state, MLE-12 cells were treated with desipramine under the indicated conditions and analyzed by RT-qPCR. Expression of epithelial markers (*Sftpc, Krt8, Ager,* and *Hopx*) and mechanosensitive genes (*Cyr61* and *Ccn2*) was evaluated following treatment. Desipramine did not induce a uniform transcriptional response across these readouts, and marker expression remained variable between replicates. Data are presented as individual replicates with the horizontal line representing the mean ± SD of n = 5 independent biological experiments. Statistical analysis was performed using an unpaired Student's t-test comparing the desipramine group to its corresponding vehicle control. These findings support the interpretation that the partial benefit observed in the WI-38/MLE-12 alveolosphere assay is unlikely to be explained by a strong epithelial cell-autonomous effect. Abbreviations: DMI, desipramine; veh, vehicle control; RT-qPCR, reverse transcription-quantitative polymerase chain reaction; h, hours; TGF-β, transforming growth factor-beta.
